# *In vivo* evaluation of drug dialyzability in a rat model of hemodialysis

**DOI:** 10.1371/journal.pone.0233925

**Published:** 2020-06-12

**Authors:** Masaki Fukunaga, Daisuke Kadowaki, Mika Mori, Satomi Hagiwara, Yuki Narita, Junji Saruwatari, Ryota Tanaka, Hiroshi Watanabe, Keishi Yamasaki, Kazuaki Taguchi, Hiroki Ito, Toru Maruyama, Masaki Otagiri, Sumio Hirata

**Affiliations:** 1 Graduate School of Pharmaceutical Sciences, Kumamoto University, Kumamoto, Japan; 2 Faculty of Pharmaceutical Sciences, Sojo University, Kumamoto, Japan; 3 DDS Research Institute, Sojo University, Kumamoto, Japan; 4 Department of Clinical Pharmacy, Oita University Hospital, Yufu, Japan; 5 Keio University Faculty of Pharmacy, Minato-ku, Japan; International University of Health and Welfare, School of Medicine, JAPAN

## Abstract

It is important to calculate the drug removal by hemodialysis (HD) for drug dosing regimens in HD patients. However, there are limited and inconsistent information about the dialyzability of drugs by HD. Therefore, the aim of our study is to evaluate drug removal by utilizing a rat model of HD (HD rat) and to extrapolate this result to the drug removal rate in HD patients. HD rats received bilateral nephrectomy and HD for 2 h. The dialysis removal of 6 drugs was evaluated in HD rats. Dialysis efficiency, plasma protein binding rate (PBR) and distribution volume (Vd) of drugs were also measured. Furthermore, we examined the correlation between the dialyzability of drug in HD rats and humans and constructed the prediction formula of the drug dialyzability in HD patients. The clearance of urea and creatinine and normalized dialysis dose in HD rats were 0.83 ± 0.07 mL/min, 0.70 ± 0.08 mL/min, and 0.13 ± 0.06, respectively. The drug dialyzability in HD rats was similar to reported clinical data except for doripenem. A higher correlation was observed between drug dialyzability in reported clinical data and HD rats which were adjusted for PBR (r^2^ = 0.936; *p* < 0.001) compared to unadjusted (r^2^ = 0.812; *p* = 0.009). Therefore, we constructed the prediction formula of the drug dialyzability in HD patients by utilizing the HD rat model and PBR. This study is useful for evaluating the dialyzability of high-risk drugs in a clinical setting and might provide appropriate preclinical dialyzability data for new drug.

## Introduction

Drug excretion by the kidney in hemodialysis (HD) patients may be altered and can be unpredictable. Therefore, it is important to calculate the drug removal for drug dosing regimens in HD patients. However, information about the dialyzability of prescribed drugs in these patients is lacking due to the discordance in the HD conditions. In addition, as the redistribution of the drug from tissue to plasma is observed in some drugs such as vancomycin (VCM) after HD (rebound phenomenon), it is difficult to make an accurate assessment of the drug dialyzability by using only the change of drug concentration before and after HD (dialyzer clearance of drugs). Therefore, optimal medication dosing in HD patients cannot be performed and this may lead to inappropriate treatment and increased risks of side effects [1, 2].

In addition to clinical studies, other studies have used *in vitro* and *ex vivo* models for investigation of drug dialyzability. However, these models do not reflect biological factors, such as Vd [3], and the drug dialyzability was not measured accurately. In contrast, *in vivo* models reflect the effect of these biological factors. However, many *in vivo* models including large animals such as dogs [4] and goats [5] are not suitable for evaluating drug dialyzability. Furthermore, PBR and Vd [6–8] and its effect on the dialyzability of drugs [9, 10] can differ among species, and this is problematic when modelling human disease in *in vivo*.

Here, the aim of our study is to establish an *in vivo* model for the evaluation of drug dialyzability, using a one-hundredth scale dialyzer, in a rat model to construct the prediction formula of drug dialyzability in HD patients.

## Material and methods

### Chemicals and reagents

**Fig 1:**
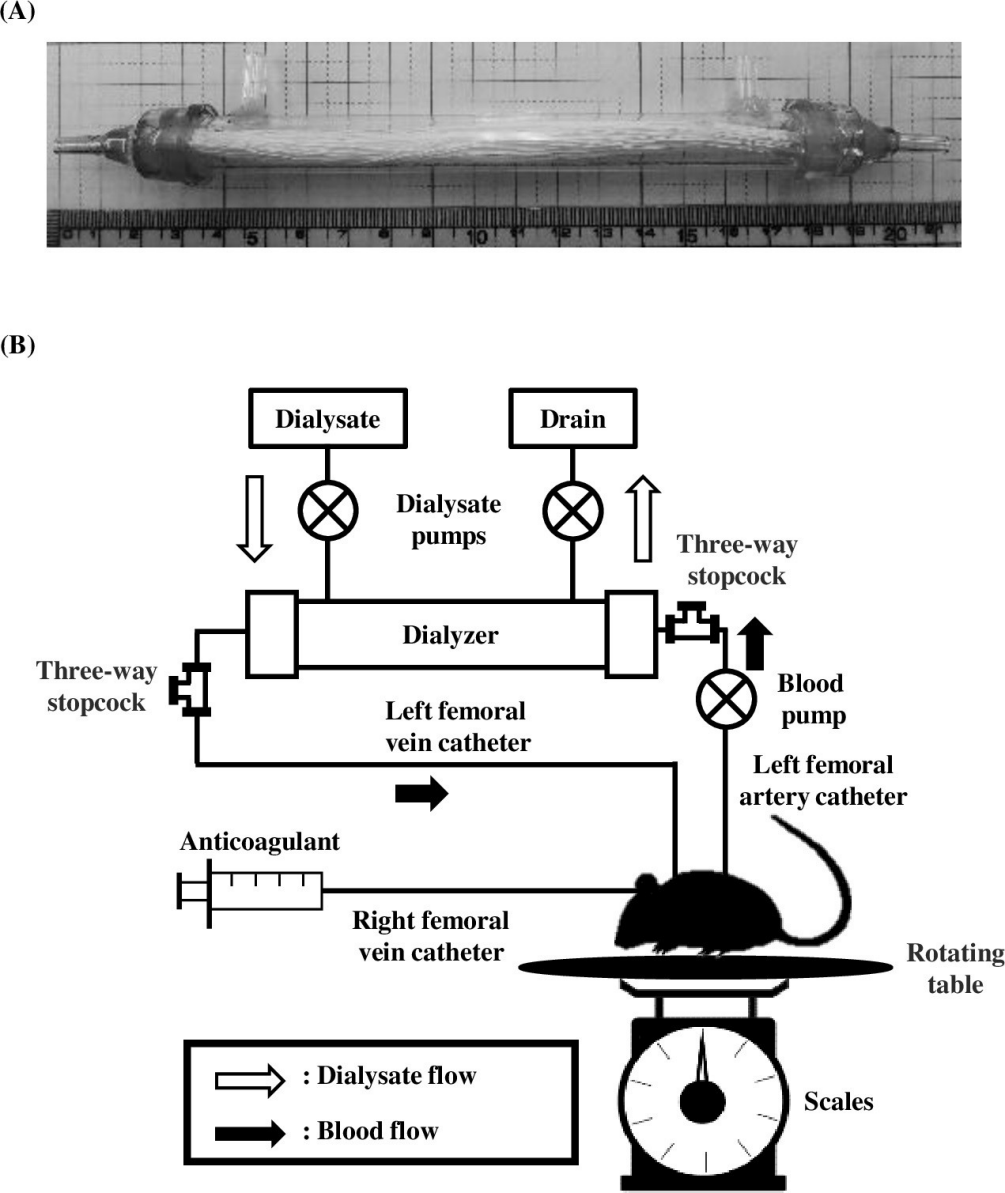
Construction of hemodialysis system using rat. The miniaturized dialyzer (A) and schematic diagram of the HD protocol (B) were shown. Miniature dialyzer contained PES membrane (113 cm^2^) and was gamma sterilized. The renal failure model rat was prepared by bilateral renal nephrectomy. Heparin (100 U/mL) was administered as an anticoagulant with 0.2 mL/h flow rate. Blood pump was AC-2120 PERISTA^®^ BIO-MINIPUMP. Dialysate pumps were AC-2120 PERISTA^®^ BIO-MINIPUMP (inflow side) and Ceramic pump VSP-2050 (outflow side).

Amikacin sulfate (AMK; AMIKACIN Sulfate Injection 100 mg “*Nichiiko*”) was purchased from Nichi-Iko Pharmaceutical Co., Ltd. (Toyama, Japan). Aprindine hydrochloride (AP; Aspenon^®^ for *iv* inj. 100) was purchased from Bayer Yakuhin, Ltd. (Osaka, Japan). Vancomycin hydrochloride (VCM; Vancomycin) and Doripenem Hydrate (DRPM; FINIBAX^®^) were purchased from SHIONOGI & Co., Ltd. (Osaka, Japan). Sodium 2-propylvalerate (VPA) was purchased from Tokyo Chemical Industry Co., Ltd. (Tokyo, Japan). Acetaminophen (APAP) was purchased from Sigma-Aldrich (St. Louis, MO., USA). Bicarbonate buffer (Sublood-BSG) and physiological saline were purchased from Fuso Pharmaceutical Industries, Ltd. (Osaka, Japan) and Heparin sodium (Heparin sodium 5,000 units/5 mL for Inj. MOCHIDA) was purchased from MOCHIDA PHARMACEUTICAL Co., Ltd. (Tokyo, Japan). Cilastatin sodium salt was purchased from Wako Pure Chemical Industries, Ltd. (Osaka, Japan). I-STAT^®^ cartridge EC8+ was purchased from Abbot Japan Co., Ltd. (Chiba, Japan). The miniaturized polyethersulfone (PES) dialyzer was provided by the Artificial Organ Development Center of the Nipro Research and Development Laboratory (Shiga, Japan). The dialyzer was shown in Fig 1 A. All other chemicals were of the highest grade and obtained from commercial sources.

### Animals

Male Wistar rats (500–800 g) were purchased from Kyudo Co. Ltd. (Saga, Japan). The study was approved by the institutional animal experiment committee at Kumamoto University (Protocol number: A29-062). The rats used in the experiments were given *ad libitum* access to ordinary laboratory chow (CE-2, CLEA Japan, Inc., Tokyo, Japan) and water, and maintained in 24 ± 1°C with a regular 12-h light-dark cycle. All surgeries were performed under three types of mixed anesthetic agents (0.15 mg/kg of medetomidine, 2.0 mg/kg of midazolam, and 2.5 mg/kg of butorphanol), with all efforts made to minimize animal suffering. During experimental periods, animals were monitored 3–4 times per day for potential signs of suffering, mainly weight loss of more than 20% and significant changes in animals’ behavior, body posture or respiration. Rats with signs of suffering or observed for 72 h following the operation were euthanized by intraperitoneal administration of pentobarbital (150 mg/kg) in order to prevent further suffering.

### Establishment of the HD rat model

A schematic diagram of the HD protocol and HD conditions were shown in Fig 1B and Table 1, respectively. Under anesthesia, a bilateral nephrectomy was carried out where the right kidney was removed by incising the flank of the rat and ligating the right renal artery, vein and ureter. After the right kidney was removed, the left kidney was also removed in the same manner.

**Table 1:** Hemodialysis conditions of HD rats compared with clinical setting.

	HD rats	Clinical condition
Vascular access	Catheter	Arteriovenous fistula ^a^ [11]
Flow rate		
Blood flow rate (mL/min)	1.0	180–239 ^a^ [11]
Dialysate flow rate (mL/min)	5.0	500–549 ^a^ [11]
Dialysis time (h)	2	3.0–5.4 ^b^ [11]
Dialyzer		
Membrane type	PES	PS, CTA, PES ^a^ [11]
Membrane area	113 cm^2^	1.2–2.1 m^2^ ^a^ [11]
Dialysate	Bicarbonate dialysate	Bicarbonate dialysate
Anesthesia	No	No

^a^ Vascular access, blood flow rate, dialysate flow rate, membrane type and membrane area at clinical condition was more than 10% of the total population in the paper.

^b^ Dialysis time at clinical condition was more than 5% of the total population in the paper.

PS: polysulfone, CTA: cellulose triacetate, PES: polyethersulfone.

The skin around the femur was incised and polyethylene tube catheters (PE No. 50, Becton, Dickinson and Company., Franklin Lakes, NJ, USA), filled with heparinized saline (100 IU/mL), were indwelled in the left femoral artery, left femoral vein, and right femoral vein. The opposing ends of the catheters were exposed at the base of the neck under the skin.

The indwelling catheters were connected to the blood circuit and the conscious rat underwent HD for 2 h, 18 h following the bilateral nephrectomy. The indwelling catheters of the left femoral artery and vein were connected to the three-way stopcocks of the blood circuit to collect the blood during HD. The indwelling catheter of the right femoral vein was used as a route of heparin administration during HD. The flow rate of the blood circuit and the dialysis fluid circuit were controlled by roller-pumps, with the blood flow and dialysate flow maintained at 1 mL/min and 5 mL/min, respectively. Sublood-BSG (bicarbonate buffer) was heated to 37°C and was used as the dialysis fluid prior to HD. The blood circuit was filled with Sublood-BSG before being filled with heparinized saline (100 IU/mL) prior to HD.

### Measurement of physiological parameters and dialysis efficiency

Blood samples were collected at 0, 18, 20, 26, 32, 44 h after rats received bilateral nephrectomy. Physiological parameters [blood urea nitrogen (BUN), sodium (Na^+^), potassium (K^+^), chroride (Cl^-^), hematocrit (Hct), hemoglobin (Hb)] in blood were measured by i-STAT 1^®^ analyzer.

The HD clearance of urea and creatinine (Cr), normalized dialysis dose (Kt/V), were also calculated as the indices of dialysis efficiency. The HD clearance of urea and Cr were calculated by sampling blood from the inlet and outlet of the dialyzer 1 h after the initiation of HD and measuring BUN and Cr using i-STAT 1^®^ analyzer and LabAssay^TM^ Creatinine (Wako Pure Chemical Industries, Ltd., Osaka, Japan). In addition, the outlet concentration of the dialyzer was adjusted by Hct in order to eliminate the influence of enrichment and dilution by dialysis. The HD clearance of urea and Cr were calculated as follows:

$$HD\;Clearance\;(mL/min)=\frac{C_{a}-C_{v}}{C_{a}}\times Q_{B}\times \left(1-Hct\right)$$

where Ca corresponds to the concentration at the inlet of the dialyzer, Cv is the concentration at the outlet of the dialyzer, Q_B_ is blood flow, and Hct is hematocrit.

Furthermore, Kt/V was calculated, based on the Daugirdas formula [12], as follows:

$$\text{Kt}/\text{V}=-\text{ln}\left(\frac{{\text{BUN}}_{\text{post}}}{{\text{BUN}}_{\text{pre}}}–\;0.008\times \text{Td}\right)+\;\left(4-3.5\times \frac{{\text{BUN}}_{\text{post}}}{{\text{BUN}}_{\text{pre}}}\right)\times \frac{\text{UFV}}{\text{BW}}$$

where BUN_pre_ and BUN_post_ are pre-dialysis and post-dialysis BUN concentration, Td is dialysis time, UFV is ultrafiltration volume, and BW is post-dialysis weight.

### Evaluation of plasma concentration-time curve of drugs and drug removal by HD

The blood concentration and the dialysis removal rate of AMK, AP, VCM, DRPM, VPA and APAP were evaluated. The drugs were continuously infused (ci) with a microsyringe pump IC 3100 (Kd Scientific Inc., Holliston, Mass., USA) via the right femoral vein catheter. The dosing rates for the drugs were as follow: 7 mg/kg/30 min (AMK), 7 mg/kg/10 min (AP), 10 mg/kg/1 h (VCM), 60 mg/kg/30 min (DRPM), 10 mg/kg/15 min (VPA) and 10 mL/kg/15 min (APAP). As the activity of the degradation of DRPM by dihydropeptidase-I (DHP-I) was higher in rat than in human [6], a DHP-I inhibitor was used in combination with DRPM.

Blood was collected chronologically after continuous *iv* injection of the drugs to evaluate the change in drug concentration in blood. The concentration of drug in the dialysate recovery fluid was calculated using the dialysate recovery volume and the drug concentration in dialysate. Furthermore, as HD was only performed for 2 h, the estimated drug removal rate of 4 h was calculated using first-order kinetics. The drug removal rate was calculated by following equation based on previous clinical studies [13, 14]:

$$drug\;removal\;rate\left(\%\right)=\frac{drug\;concentration\;in\;dialysate\left(mg/mL\right)\times dialysate\;recovery\;volumemL}{drug\;dosemg}\times 100$$

Clinical data of drug dialyzability were quoted from previous reports.

AMK was measured using TDX^TM^ amikacin “Abbott” with a fluorescence polarization immunoassay method. AP, VCM, DRPM, VPA, and APAP were measured by HPLC. The HPLC instrumentation for AP consisted of an LC-10AS pump (Shimadzu GLC) and an SPD-10A UV detector (Shimadzu GLC). The HPLC instrumentation for other drugs consisted of an Alliance®2695 HPLC device (Waters Corporation, Milford, Mass., USA) and a 2489 UV/Vis detector (Waters Corporation, Milford, Mass., USA). The measurement of AP, DRPM, APAP and VPA was based on previously reported methods [15–18]. VCM samples were prepared in 40 μL of sample serum, with 10 μL of 50 mM 1H-benzotriazole as an internal standard and 40 μL of methanol. This was then vortexed for 30 sec and centrifuged at 18000 g for 5 min at 4°C. The supernatant of each sample was analyzed using COSMOSIL 5C18-MS-II Packed Column (5 μm, Ф4.6 × 250 mm, Nacalai Tesque Inc., Kyoto, Japan) and acetonitrile-20 mM sodium phosphate buffer (the ratio of acetonitrile to buffer, 12:88). The flow rate of the mobile phase was 1 mL/min and the column temperature was kept at 25°C. The detection wavelength was 210 nm and the injection volume was 25 μL.

### PBR and Vd in human and rat

The serum of rat 18 h after bilateral nephrectomy and HD patients were used for evaluating PBR. The use of serum obtained from HD patients was approved by the clinical research review board at Kumamoto University (approved number: 1578). All HD patients provided written informed consent. After the drug was added to the serum, it was placed in a sample reservoir and centrifugal filtration was performed. The ultrafiltration of VCM samples were performed with Vivaspin 500 (NIPPON Genetics Co., Ltd., Tokyo, Japan), and Amicon Ultra-0.5 (Merck Millipore Massachusetts, USA) was used for other samples. The concentration of drug in the serum and ultrafiltrate was measured to calculate the PBR.

The PBR was calculated by the following equation:

$$\text{PBR}\;\%=\frac{{\text{C}}_{\text{T}}-{\text{C}}_{\text{F}}}{{\text{C}}_{\text{T}}}\times 100$$

where C_T_ was the drug concentration of the serum before ultrafiltration and C_F_ was the drug concentration of the serum in ultrafiltrate.

A non-compartment model was used for the pharmacokinetic analysis. Each parameter was calculated using the moment analysis program available on Microsoft Excel. Vd of drugs in humans were quoted from previous reports.

### Statistics

The data were expressed as mean ± standard deviation (SD). The statistical analyses were performed using the Statcel4 software (OMS publishing Inc., Saitama, Japan). Statistical significance was evaluated using a one-tailed paired *t*-test for single comparison or two-tailed student's *t*-test for comparisons between two means. In addition, we investigated the association between drug dialyzability in humans (DD_human_) and drug removal rate in HD rats, while carefully considering other pharmacokinetic parameters, e.g., PBR and Vd in rats and humans, using single or multiple linear regression analysis. We evaluated the equations described below and selected the prediction formula based on the adjusted square of correlation coefficient. This analysis was carried out using the R software program (version 3.0.0; R Foundation for Statistical Computing, Vienna, Austria). A *p* less than 0.05 was considered statistically significant.

$${\text{DD}}_{\text{human}}=a_{0}\;+a_{1}\;\times \;\text{drug}\;\text{removal}\;\text{rate}\;\text{in}\;\text{HD}\;\text{rat,}$$ (1)

$${\text{DD}}_{\text{human}}=a_{0}\;+a_{1}\;\times \;\text{drug}\;\text{removal}\;\text{rate}\;\text{in}\;\text{HD}\;\text{rat}\;\times \;\text{plasma}\;\text{protein}\;\text{unbinding}\;\text{rate}\left({\text{PBR}}_{\text{unbind}}\right)\left(\text{human}\right){\text{/PBR}}_{\text{unbind}}\left(\text{rat}\right),$$ (2)

$${\text{DD}}_{\text{human}}=a_{0}\;+a_{1}\;\times \;\text{drug}\;\text{removal}\;\text{rate}\;\text{in}\;\text{HD}\;\text{rat}\;+a_{2}\;\times \;\text{Vd}\;\left(\text{human}\right)\;/\;\text{Vd}\;\left(\text{rat}\right),$$ (3)

$${\text{DD}}_{\text{human}}=a_{0}\;+a_{1}\;\times \;\text{drug}\;\text{removal}\;\text{rate}\;\text{of}\;\text{HD}\;\text{rat}\;+a_{2}\;\times \;[{\text{PBR}}_{\text{unbind}}/\;\text{Vd}\;\left(\text{human}\right)]\;/\;[{\text{PBR}}_{\text{unbind}}/\;\text{Vd}\;\left(\text{rat}\right)],$$ (4)

where *a*_0_ is the constant; *a*_1_ and *a*_2_ are the coefficients of parameters.

## Results

### Measurement of physiological parameters and dialysis efficiency in HD rats

**Fig 2:**
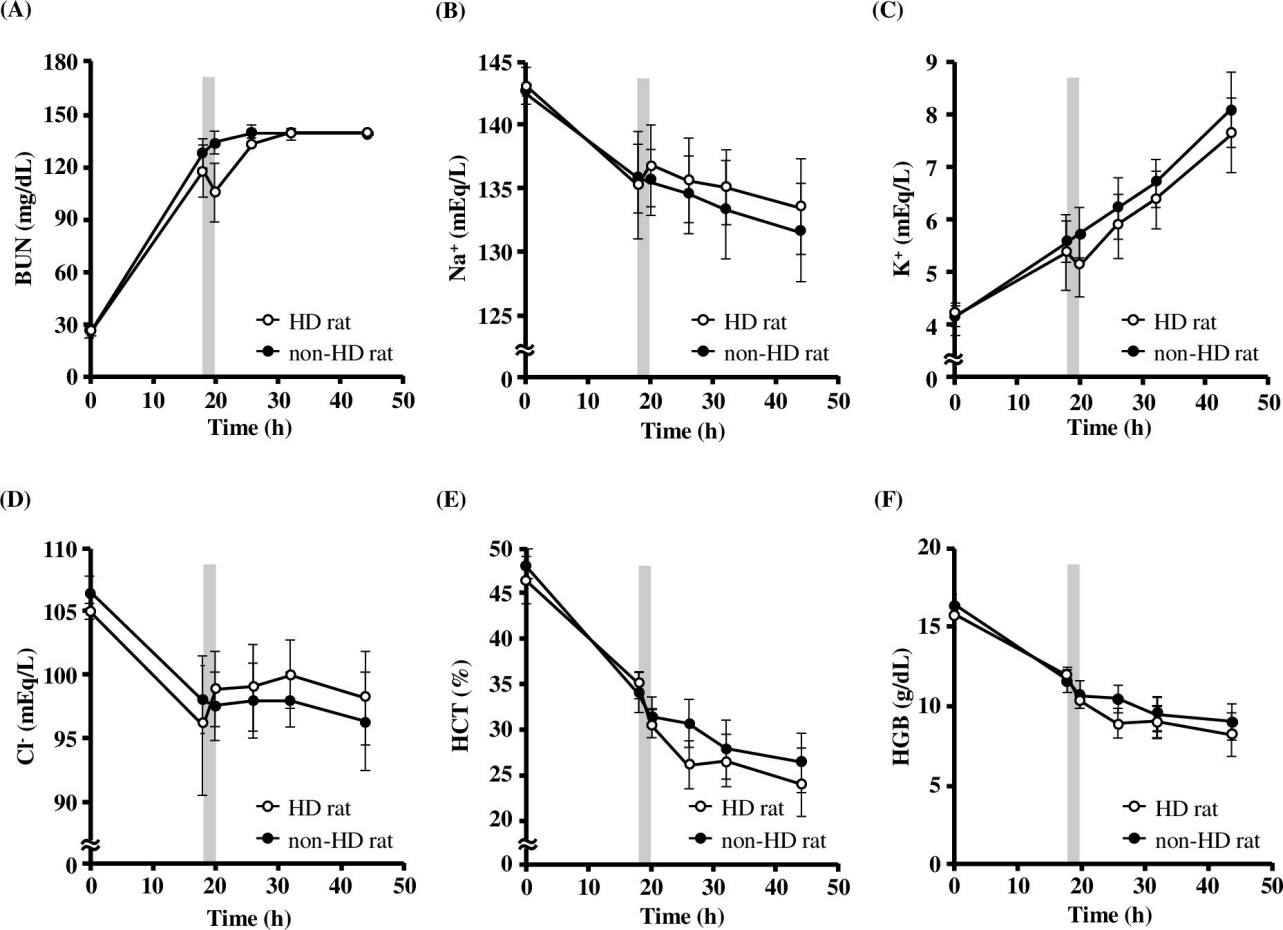
Physiological parameters of each rat with or without HD. The blood of each HD rat was corrected and BUN (A), Na^+^ (B), K^+^ (C), Cl^-^ (D), Hct (E) and Hb (F) were measured. Hemodialysis timing was represented by the gray area. The value of BUN reached a plateau at 140 mg/dL, which is the measurement limit of the i-STAT^®^ 1 Analyzer. ○: HD rat (*n* = 4–7), ●: non-HD rat (*n* = 3–4). Values are expressed as the mean ± SD. BUN: blood urea nitrogen, Na^+^: sodium, K^+^: potassium, Cl^-^: chloride, Hct: Hematocrit, Hb: Hemoglobin.

The plasma concentration-time curve of physiological parameters in HD rats were shown in Fig 2. HD rats were compared to rats not undergoing HD (non-HD rats). Both models showed an increase in BUN and K^+^ and a decrease in Na^+^, Cl^-^, Hct and Hb by 18 h after the bilateral nephrectomy. HD rats showed an increase in Na^+^ and Cl^-^ and a decrease in BUN and K^+^ during HD, with physiological parameters corrected in comparison to non-HD rats (Fig 2A–2D). On the other hand, Hct and Hb decreased over time (Fig 2E and 2F). In addition, body weight of HD rats did not change significantly before and after HD (before HD: 660.8±77.2 g, after HD: 658.4±76.3 g). The dialysis efficiency in HD rats was shown in Table 2. The HD clearance of urea, Cr and Kt/V in HD rats were 0.83 ± 0.07 mL/min, 0.70 ± 0.08 mL/min, and 0.13 ± 0.06. respectively.

**Table 2:** Dialysis efficiency by HD rats.

	CL_Urea_ (mL/min)	CL_Cr_ (mL/min)	Kt/V
*In vivo* model	0.83 ± 0.06	0.70 ± 0.08	0.13 ± 0.06
Clinical condition	> 150 ^a^ [19]	> 130 ^a^ [19]	0.9 ~1.9 ^b^ [11]

^a^ CL_Urea_ and CL_Cr_ at clinical condition were the lower limit of the performance standard required for functional classification of dialyzer with a membrane area of 1.5 m^2^ based on clinical data.

^b^ Kt/V at clinical condition was more than 5% of the total population in the paper.

Values are expressed as the mean ± SD.

CL_Urea_ and CL_Cr_: the HD clearance of urea and creatinine, Kt/V: normalized dialysis dose.

### Evaluation of drug removal by HD rats

**Fig 3:**
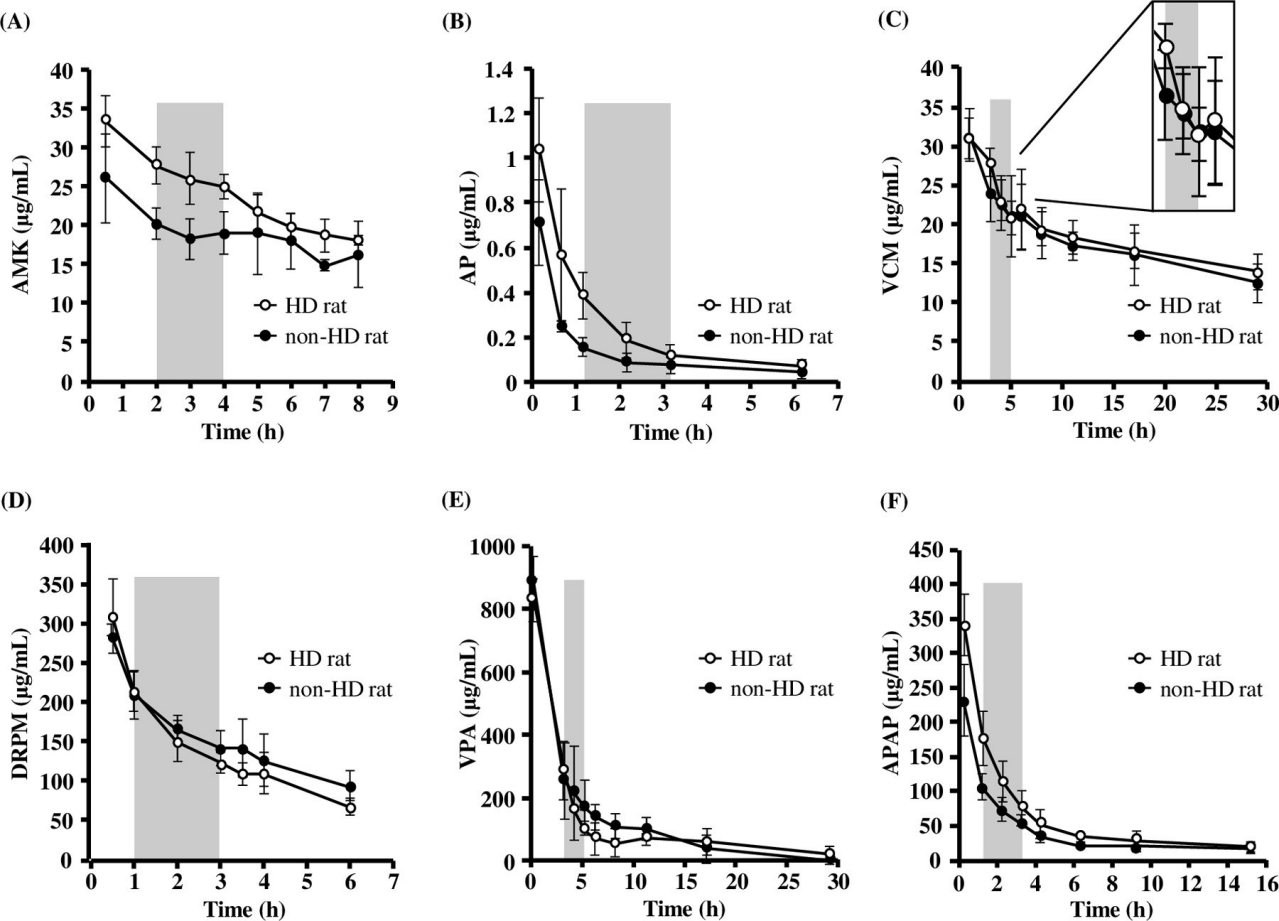
Plasma concentration-time curve of drugs by HD. The blood of each HD rat was corrected and concentration of drugs [AMK (A), AP (B), VCM (C), DRPM (D), VPA (E) and APAP (F)] was measured. Hemodialysis timing was represented by the gray area. ○: HD rat (*n* = 4–7), ●: non-HD rat (*n* = 3–4). Values are expressed as the mean ± SD. AMK: amikacin sulfate, AP: aprindine hydrochloride, VCM: vancomycin hydrochloride, DRPM: doripenem hydrate, VPA: sodium 2-propylvalerate (valproic acid sodium), APAP: acetaminophen.

The evaluation of plasma concentration-time curve and dialysis removal rate of AMK, AP, VCM, DRPM, VPA, and APAP were performed using HD rats. The plasma concentration-time curve of each drug in HD rats, as compared to non-HD rats, was shown in Fig 3. The plasma concentration of each drug was decreased. However, this was not the case for VCM as, in regard to the rebound phenomenon, there was a rapid increase in plasma concentration after HD. Measured drug removal rate in HD rats, with estimated drug removal rate at 4 h, and reported clinical data were shown in Table 3. The majority of the estimated drug removal rates were similar to reported clinical data, except for DRPM. The estimated drug removal rate observed lower dialyzability of DRPM than reported clinical data. (HD rats vs. reported clinical data; 29.6 ± 3.2% vs 46.3–56.1%).

**Table 3:** Comparison of drug removal of various drugs between humans and *in vivo* rat model.

	Rats (%)		Humans (%)
Hemodialysis time	2 h	4 h (estimated)	4 h
AMK	27.1 ± 2.7	46.8 ± 4.0	53 [13]
AP	None^a^	0	None^a^ [10]
VCM	17.8 ± 3.1	32.4 ± 5.1	20.8–39.5 [20–22]
DRPM	16.1 ± 1.9	29.6 ± 3.2	46.3–56.1^b^ [23]
VPA	8.4 ± 3.7	15.9 ± 6.6	15.1–21.9 [24, 25]
APAP	6.1 ± 0.8	11.8 ± 1.5	10.8 [14]

Estimated drug removal rate of 4 h was calculated using first-order kinetics.

^a^ AP was not detected in dialysate.

^b^ Some data on removal rate of DRPM were obtained from SHIONOGI & Co., Ltd.

Values are expressed as the mean ± SD.

AMK: amikacin sulfate, AP: aprindine hydrochloride, VCM: vancomycin hydrochloride, DRPM: doripenem hydrate, VPA: sodium 2-propylvalerate (valproic acid sodium salt), APAP: acetaminophen.

### The difference in PBR and Vd between human and rat

The PBR and Vd of each drug in rats and humans were shown in Table 4. Rats had higher PBR of DRPM than humans (rats vs. humans; 26.1 ± 4.2% vs 2.5 ± 2.2%, *p* < 0.001). The Vd of VCM, DRPM and VPA were similar. Whereas AMK, AP and APAP were not similar between rats and humans (rats vs humans: AMK; 0.71 ± 0.18% vs 0.35–0.39%, AP: 54.5 ± 15.3% vs 4.1–10.5%, APAP: 24.4 ± 7.4% vs 4.59%).

**Table 4:** PBR and Vd of various drugs between humans and *in vivo* rat model.

	PBR (%)		Vd (L/kg)	
	Rats	Humans	Rats	Humans
AMK	0.8 ± 6.2	0.5 ± 5.7	0.71 ± 0.18	0.35–0.39 [26, 27]
AP	93.3±1.0	91.2±1.2	54.51 ± 15.33	4.1–10.5^a^ [28]
VCM	35.0 ± 9.3	35.3 ± 6.9	0.85 ± 0.11	0.51–0.94 [20, 21]
DRPM	26.1 ± 4.2**	2.5 ± 2.2	0.37 ± 0.05	0.30–0.40^b^ [23, 29]
VPA	24.4 ± 7.4	38.9 ± 5.0	0.46 ± 0.18	0.1–0.4 [10, 30]
APAP	25.7 ± 3.1	24.0 ± 1.7	2.21 ± 0.96	3.18^c^ [31]

^a^ Vd were calculated by dividing Vd at steady state by the average of weight

^b^ Some Vd was calculated by dividing the average of Vd at steady state by the average of weight

^c^ Vd were calculated by using half-life, the area under the curve, APAP dose and weight of HD patients. It was postulated that the bioavailability of APAP was 100%.

Values are expressed as the mean ± SD.

PBR: protein binding rate, Vd: distribution volume, AMK: amikacin sulfate, AP: aprindine hydrochloride, VCM: vancomycin hydrochloride, DRPM: doripenem hydrate, VPA: sodium 2-propylvalerate (valproicacid sodium salt), APAP: acetaminophen.

**p <0.01 compared with humans.

### Predictive equations of drug removal by HD patients using HD-rat parameters

We developed the HD-rats parameters-based predictive equation for drug dialyzabilities in HD patients using single or multiple linear regression model (Table 5).

**Table 5:** Parameter of predictive equation for drug dialyzabilities in HD patients using single or multiple linear regression model.

Parameter	*a*_0_	*a*_1_	*a*_2_	*P value*
Observed Eq 1	1.412	1.264	-	0.009
Observed Eq 2	0.9013	1.227	-	< 0.001
Observed Eq 3	1.440	1.264	-0.02775	0.058
Observed Eq 4	2.868	1.235	-0.3877	0.058

*a*_0_ is the constant; *a*_1_ and *a*_2_ are the coefficients of parameters.

**Fig 4:**
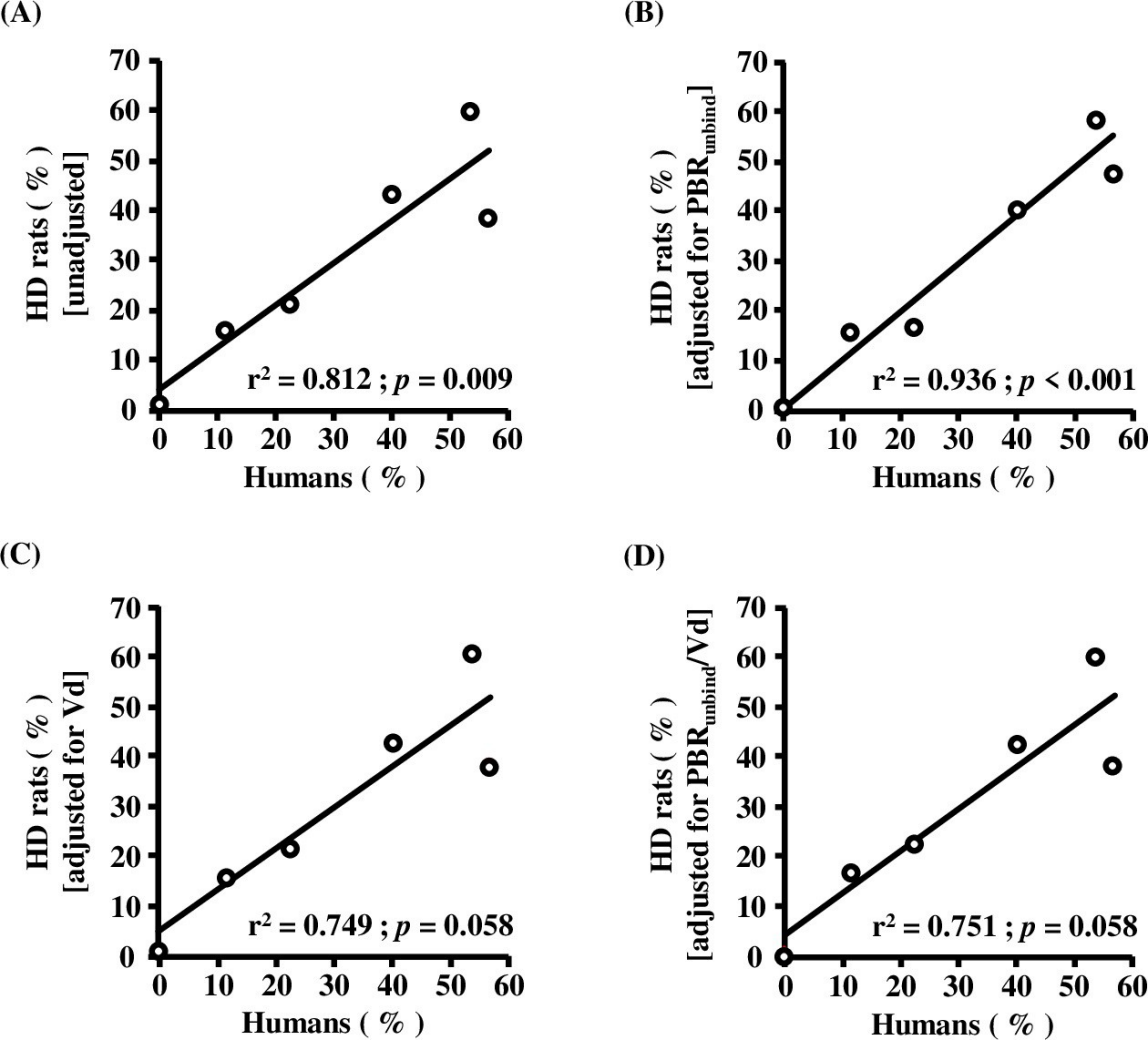
Relationship between drug removal rates in humans and HD rats adjusted by various drug characteristics. Relationship between drug removal rates in humans and unadjusted HD rats (A), HD rats adjusted for PBR_unbind_ (B), HD rats adjusted for Vd (C), HD rats adjusted for PBR_unbind_/Vd (D). Correlativity between drug removal rates in humans and HD rats adjusted by various drug characteristics was calculated by R software program. PBR_unbind_: protein unbinding rate, Vd: distribution volume.

We examined the association between drug dialyzabilities of the 6 drugs in clinical data reported previously and those observed in HD rats (Fig 4). The adjusted squares of correlation coefficient were 0.812, 0.936, 0.749 and 0.751in observed Eqs 1, 2, 3 and 4 respectively. Therefore, we selected the observed Eq 2, in which the drug dialyzability in HD patients were estimated based on that in the HD rat model and PBR_unbind_, as the best predictive formula.

## Discussion

We established an *in vivo* model of HD in rats for evaluation of drug dialyzability. There have been studies on *in vitro* drug clearance of dialyzer [3, 32]. However, because *in vitro* study cannot reflect Vd and drug clearance usually assesses at only one point, it is difficult to evaluate the prediction of drug dialyzability using *in vitro* drug clearance. On the other hand, HD rat which can reflect Vd and evaluate drug removal rate is higher predictability of drug removal than *in vitro* drug clearance of dialyzer. Moreover, several previous studies have shown that *in vivo* HD models were used for evaluating the dialyzer performance or the change of physiological parameters in HD [4, 5, 33–36], but the drug dialyzability using *in vivo* model has never been reported. Therefore, this is the first study to evaluate drug dialyzability using *in vivo* model and extrapolate the drug dialyzability from *in vivo* model to human. On the benefits of this model, HD rat more reflects clinical condition than previous studies [4, 5, 33–35], such as the use of high-performance membrane dialyzer, HD underwent with non-anesthesia, and the use of bicarbonate buffer as dialysate. Furthermore, there have been some report on HD model by large animal [4, 5], but feeding of large animal models has higher costs than rats, and it is hard to operate large animal. In addition, the cost of HD in large animal is very expensive. These problems will overcome by the use of HD rat.

Bilateral nephrectomized rats were used for eliminating complete drug excretion from the kidney in order to examine drug removal by HD. Decline in kidney function, electrolyte abnormalities, and anemia were observed 18 h post bilateral nephrectomy. This was also seen in rats that were anuric [37], and this model reflected the pathology of HD patients. HD rats also removed uremic substances and corrected electrolyte abnormalities in comparison with non-HD rats (Fig 2), which is the role of HD in the clinical setting. The HD clearance of urea and Cr were approximately 1/200 of clinical setting, respectively (Table 2). As blood flow rate (1 mL/min in this model) is strongly related to HD clearance, which is 1/200 of the clinical condition, the HD clearance is 1/200 in this study. Furthermore, Kt/V was approximately 1/10 of clinical setting (Table 2). Considering Daugirdas formula [12], there are several reasons for small Kt/V in the model compared to that under the clinical condition, such as shorter duration of the modelled HD condition and less fluid removal per body weight. Another important factor was that the ratio of BUN before and after HD (BUN_post_/BUN_pre_) was higher in HD rats. HD rats had higher BUN at the beginning of HD, and the BUN reduction rate by HD was smaller (pre-dialysis BUN: 117 mg/dL, post-dialysis BUN: 106 mg/dL) compared to that in a previous clinical report [38]. On the other hand, BUN reduction rate at the inlet and outlet of the dialyzer 1 h after the initiation of HD was similar to that in the clinical report (BUN at the inlet: 111 mg/dL, BUN at outlet: 18 mg/dL) [38]. Therefore, it can be concluded that the miniaturized dialyzer in HD rats showed a performance similar to that in the clinical dialyzer. However, BUN was not as efficiently removed and the ratio of BUN_post_/BUN_pre_ was higher due to high BUN levels at the beginning of HD, short duration of HD, and slow blood flow rate [39, 40]. Furthermore, a decrease in Kt/V may be affected by BUN generation related to the species differences and feeding.

Five out of six drugs had similar dialyzability between HD rats and clinical data. The rebound phenomenon of VCM occurring after HD in the clinical setting [41, 42] was also observed in HD rats. On the other hand, it is considered that the lower dialyzability of DRPM observed in HD rats was due to higher PBR of DRPM in rat compared with human (Table 4). Furthermore, the rats that underwent HD were not anesthetized, whereas some of the HD animal models reported have undergone HD under anesthesia [4, 36]. In fact, the presence or absence of anesthesia affected the drug dialyzability and the non-anesthesia HD model tended to reflect more of the reported clinical data on the dialyzability of VCM (20.8–39.5%) than anesthesia HD model in our previous study (the non-anesthesia HD model vs the anesthesia HD model; 32.4 ± 5.1% vs 41.9 ± 9.2%). Therefore, it is suggested that the HD rat model is useful for evaluating drug dialyzability due to reflecting reported clinical data and influencing different species PBR. Furthermore, this non-anesthesia HD rat model reflects the clinical condition.

Previously, it has been reported that there are species differences in PBR and Vd of drugs [6–8]. Thus, we calculated PBR and Vd of 6 examined drugs in both human and rat. This study observed differences in PBR and Vd between rats and humans (Table 4). Therefore, it is necessary that we consider the influence of species differences in biological factors, such as PBR, in order to extrapolate from HD rats to humans.

Therefore, we examined the correlation between 6 drug dialyzability in reported clinical data and HD rats adjusted for PBR and/or Vd. As a result, there was a correlation between drug dialyzability in reported clinical data and measured drug removal rate of HD rats (r^2^ = 0.812; *p* = 0.009) (Fig 4 A). In addition, drug dialyzability in HD rats was adjusted for PBR and/or Vd in order to make the correlation higher. A higher correlation of drug dialyzability was observed between reported clinical data and HD rats adjusted for PBR (r ^2^ = 0.936; *p* < 0.001) (Fig 4 B). This supported the results of DRPM dialyzability in HD rats. On the other hand, there was no correlation between drug dialyzability in reported clinical data and HD rats adjusted for Vd nor PBR and Vd (Fig 4C and 4D). So far, it has been reported that PBR has a higher correlation with drug dialyzability than with Vd [9, 10] and these results support this. In contrast, as AP and APAP have a large Vd and are their removal is limited by HD, it is considered that drug dialyzability is affected less by the species differences than Vd. However, it is suggested that the prediction formula of drug dialyzability in HD patients, by utilizing the HD rat model, and PBR is a very useful prediction equation considering the influence of the species differences in biological factors.

However, there were some limitations in this study. Firstly, there was an upper limit of blood flow rate and dialysis membrane area in HD rats. As rats are small in size compared with humans, an increase in blood flow rate or extracorporeal blood volume increases the burden on the rat. Therefore, it is difficult to increase blood flow rate or dialysis membrane area more than this dialysis condition and the impact of blood flow rate and membrane area on drug dialyzability will have to be assessed using *in vitro* and *ex vivo* models. Secondly, we examined a small number of drugs. Only 6 drugs were evaluated for drug dialyzability in this study, more drug dialyzability studies, using the HD rat model, will be necessary. Therefore, we will need to construct a more accurate prediction formula of the drug dialyzability by evaluating the dialyzability of various drugs such as meropenem and cefazolin in HD rats. Meropenem is reported to have a species difference involving PBR [6]. Whereas, cefazolin is reported to have a species difference involving Vd [8]. Thirdly, there are few data on drug dialyzability and pharmacokinetics in HD patients. In this study, clinical data is quoted from previous papers. However, there are few reports on drug dialyzability in the clinical setting and we cannot satisfy certain dialysis conditions such as blood flow rate, dialysate flow rate and membrane area. Some clinical data of Vd were also quoted from healthy subjects and children. Fourthly, it is important to measure both blood pressure and pressure inside the dialyzing circuit. During experimental period, we did not observe hypotensive state. We developed this HD model as a model to evaluate drug dialyzability. To our knowledge, there are no reports that blood pressure, but blood flow, could affect the dialyzability of drugs. Monitoring of blood pressure will provide more information about relationship between blood pressure and dialyzability. Therefore, we need further experiments in the future.

In this study, it is suggested that HD influenced factors associated with drug dialyzability, such as PBR. Furthermore, we can predict the drug dialyzability in HD patients by using our HD rat model and the PBR of drugs. In the future, the HD rat is a useful model for evaluating the drug dialyzability in a clinical setting and might provide the appropriate preclinical data of drug dialyzability to predict the high-risk drugs in HD patients.
